# Inhibition of Cathepsin S Restores TGF-β-induced Epithelial-to-mesenchymal Transition and Tight Junction Turnover in Glioblastoma Cells

**DOI:** 10.7150/jca.50631

**Published:** 2021-01-15

**Authors:** Li Wei, Naiyuan Shao, Ya Peng, Peng Zhou

**Affiliations:** 1 Department of Tumor Biological Treatment, the Third Affiliated Hospital of Soochow University, Changzhou, Jiangsu 213003, P.R. China.; 2 Department of Neurosurgery, The Third Affiliated Hospital of Soochow University, Changzhou, Jiangsu 213003, P.R. China.

**Keywords:** CTSS, EMT, Glioblastoma, TGF-β

## Abstract

**Background:** Invasive growth is one of the most typical features of aggressive types of malignant cancer, including glioblastoma. Lysosomal cysteine protease-cathepsin S (CTSS), has been reported to be involved in invasive growth and distant metastasis of cancer cells. However, the underlying mechanisms remained elusive.

**Methods:** U87 and U251 human glioblastoma cell lines were applied in this study. Cell migration and invasion ability were measured by wound healing assay and transwell assay. Western blot was employed to detect the expression levels of proteins. Immunofluorescence assays of cells and tissues were used to visualize the localization and expression of proteins. The SPSS software was used for statistical analysis.

**Results:** Our results showed that the high expression of CTSS was link with the grades of glioma tissues. The CTSS inhibitor-Z-FL-COCHO (ZFL), could attenuate TGF-β-induced invasive growth as proven by wound healing and transwell assays. Furthermore, inhibition of CTSS could reverse TGF-β-induced epithelial-to-mesenchymal transition (EMT) and restore TGF-β-triggered tight junction proteins turnover, thus decreasing glioblastoma cell mobility. We also observed that TGF-β could change the morphology of glioblastoma cells, redistribute intermediate-filament, vimentin, which was highly relevant to mesenchymal type cells and enhanced mobility. However, inhibition of CTSS could significantly restore this transformation. Our results proved that PI3K/AKT/mTOR pathway was significantly suppressed in the TGF-β+ZFL (CTSS inhibitor) groups, and AKT activator-SC79, could reverse the anti-invasion effect of CTSS, indicating an important role of PI3K/AKT/mTOR pathway in this process.

**Conclusion:** Z-FL-COCHO (ZFL), a CTSS inhibitor, could reverse TGF-β-induced EMT and change of tight junction proteins via PI3K/AKT/mTOR pathway.

## Introduction

Glioblastoma is the most malignant tumor among the cancer atlas of the central nervous system. The ultimate outcome of current treatment modalities is often not satisfactory in glioblastoma patients, including surgery as well as radiotherapy and chemotherapy. Latest studies showed that the progression-free survival rate and the median survival of glioblastoma patients were roughly 7 months and 15 months, respectively 1. In order to obtain a deeper understanding of characteristics of glioblastoma, studies at the cellular and molecular levels were highly above the agenda to find potential therapeutic targets.

Invasive growth is a common characteristic of malignant cancers, including glioblastoma. Dispersed glioblastoma cells around the lesion of visible tumor margin make it inevitable to relapse after massive resection. Thus, inhibition of tumor cell invasive growth would be a promising strategy to enhance the efficiency of therapeutic regime nowadays. Epithelial-to-mesenchymal transition (EMT) has emerged as an important mechanism for invasive capability of various tumors. In the context of glioblastoma, EMT related factors was significantly elevated in glioblastoma 2, and these changes were highly relevant to the grade of glioma and the prognosis of patients 3.

Transforming growth factor (TGF)-β signaling was reported to play an important role in cancer. Even though its anti-proliferation function in the normal and tumors tissue of early stage by G1 arrest and stimulation of cyclin-dependent kinase inhibitors, TGF-β acts as a tumor promotor in the late stage of different tumors, including glioblastoma^4^. Several studies have implicated that TGF-β pathway could induce EMT process in different cancers. Once binding to TGF-β receptor, TGF-β could exert its functions via Smad pathway or Smad-independent signaling pathways including PI3K/AKT, Ras/ERK, p38 kinase, and small GTPase (RHOA, PKN, Rock). Previous studies have indicated that TGF-β could maintain mesenchymal stem-like population in the GBM cells by interacting with CD44 4, 5. Additionally, during TGF-β-induced EMT, the change of the tight junction proteins was also observed.

Lysosomal cysteine proteases are universally existent in all animals and other organisms. Aberrant expression of lysosomal cysteine proteases has widely reported in numerous malignant tumors such as glioblastoma 6, 7. As functional protease in lysosome, the high expression of cathepsin family members firmly participated in the activities of autophagy-related metabolism in cancer cells. Therefore, inhibition of lysosomal cysteine proteases (for instance, CTSB, CTSD, CTSL) could contribute to autophagy disturbance, inducing apoptosis or enhancing effectiveness of anti-cancer drugs in different tumors 6, 7. However, Cathepasin S (CTSS) possesses unique roles compared with other family members. Previous studies have shown that CTSS could act as a pivotal mediator to mediate antigen presentation in major histocompatibility complex class II 8. Another distinctive feature of CTSS is the existence of out-lysosome, which was previously reported in cancer metastasis for its role of degrading extracellular matrix (ECM) proteins including laminin, fibronectin elastin, osteocalcin and some collagens 9. Till now, the effect of CTSS on EMT in glioblastoma is needed a further investigation.

Here, we provided the evidence that Z-FL-COCHO (ZFL), a type of CTSS inhibitor, could reverse TGF-β-induced EMT and the change of tight junction proteins via the PI3K/AKT/mTOR pathway.

## Materials and Methods

### Cell culture

Human glioblastoma cell lines U87 and U251 (FuHeng Cell Center, Shanghai, China) were cultured in DMEM with 1% penicillin/streptomycin (HyClone, GE Healthcare Life Sciences, Logan, UT, USA) and 10% fetal bovine serum (Thermo Fisher Scientific) at 37°C in a 5% CO2-humidified incubator.

### Regents and antibodies

TGF-β and SC-79 were obtained from Beyotime Co (Shanghai, China). Z-FL-COCHO (ZFL) was purchased from Calbiochem Co. (Darmstadt, Germany). Anti-E-cadherin (#5023), anti-ZO-1 (#13663), anti-Claudin 1 (#13995), anti-AKT (#4685), anti-p-AKT (#4060), anti-PI3k (#4249), anti-p-PI3k (#17366), anti-mTOR (#2972), anti-p-mTOR (#2971), and anti-β-actin (#4970) antibodies were purchased from Cell Signaling Technology (Danvers, MA, USA). Anti-CTSS (ab134157), anti-N-cadherin (ab18203), anti-occludin (ab216327) antibodies were purchased from Abcam (Cambridge, MA, USA). For F-actin staining, Phalloidin-iFluor 488 (ab176753) was obtained from Abcam (Cambridge, MA, USA).

### Western blot analysis

The total proteins of human glioma tissues and glioblastoma cells were extracted. The protein samples with same quality were were separated by electrophoresis and then transferred to PVDF membranes (EMD Millipore, Billerica, MA, USA). After blocked by 5% skim milk for 2 hours at room temperature, the membranes were incubated with different primary antibodies. Then, the secondary antibodies were followed next day. The blots were visualized with chemiluminescent detection kit (P90720, Millipore, MA, USA) was used to analyze the blots. The protein levels were quantified by densitometry using Image J software, and normalized to the corresponding β-Actin level.

### Migration and invasion assay

To detect the motility and invasive captivity of U87 and U251 cells under different circumstances, transwells were employed here. For migration assay, a total of 2×10^5^ cells in 200 µL DMEM were added into the upper chamber of 6.5 mm transwells with 8.0 µm pore (Corning Incorporated, Corning, NY, USA). For invasion assay, the upper chambers were covered with additional matrigel matrix (Corning Incorporated). After incubating at 37°C for 16 hours, cells in the interior of the chamber were cleaned up with cotton swab. Then, the chambers were fixed with paraformaldehyde for 15 minutes and subsequently stained with 0.1% crystal violet for 10 minutes. Six randomly selected fields were photographed under an inverted microscope (Carl Zeiss Meditec AG, Jena, Germany) at 200× magnification, and the number of stained cells was counted.

### Wound healing assay

For wound-healing assays, the cell suspensions (70 μl, 5 × 10^5^ cells/ml) of U251 or U87 were seeded into the insert of a 35 mm high culture µ-dish (Ibidi, Martinsried, Germany). After culturing for 24 h at 37 °C with 5% CO_2_, the insert was gently removed using a tweezer, the photographs of wounded areas were taken under an inverted microscope (Carl Zeiss Meditec AG, Jena, Germany) at 0 h, 24 h and 48 h, respectively. The migration distance was analyzed by Image-Pro Plus 6.0 software (Media Cybernetics, MD, USA), and the migration rate was calculated using the following formula: (1- distance at 24 h/distance at 0 h) %.

### Immunofluorescence analysis of cells and tissues

Cells were inoculated onto the circle microscope cover glass overnight and subsequent treatments were followed. Human tumor tissues were obtained from Jinling hospital under the supervision of the Ethics Committee of Nanjing Medical University. The tumor tissues were fixed, and embedded in paraformaldehyde for IHC and IF staining. Tissue sections (7 µm) were made with freezing microtome. Briefly, slides were fixed with paraformaldehyde for 15 minutes and then treated with 0.3% Triton X-100 for 10 minutes. After fetal bovine serum blocked for 30 minutes, specific primary antibodies and subsequent matched secondary antibodies were stained on the slides. Nuclei was stained with DAPI (Sigma-Aldrich, D9542, 1/2000) for 10 minutes at last. All the slides and tumor sections were observed and captured under ZEISS immunofluorescence microscope (ZEISS, German).

### Statistical analysis

The SPSS version 22.0 (IBM Corporation, Armonk, NY, USA) software was applied to analyze all the data. Data were expressed as mean ± SEM and evaluated by Student's t-test and ANOVA for multiple comparisons. P<0.05 was considered as a gauge of statistical difference.

## Results

### Cathepsin S is highly expressed in GBM tissues

**Figure 1:**
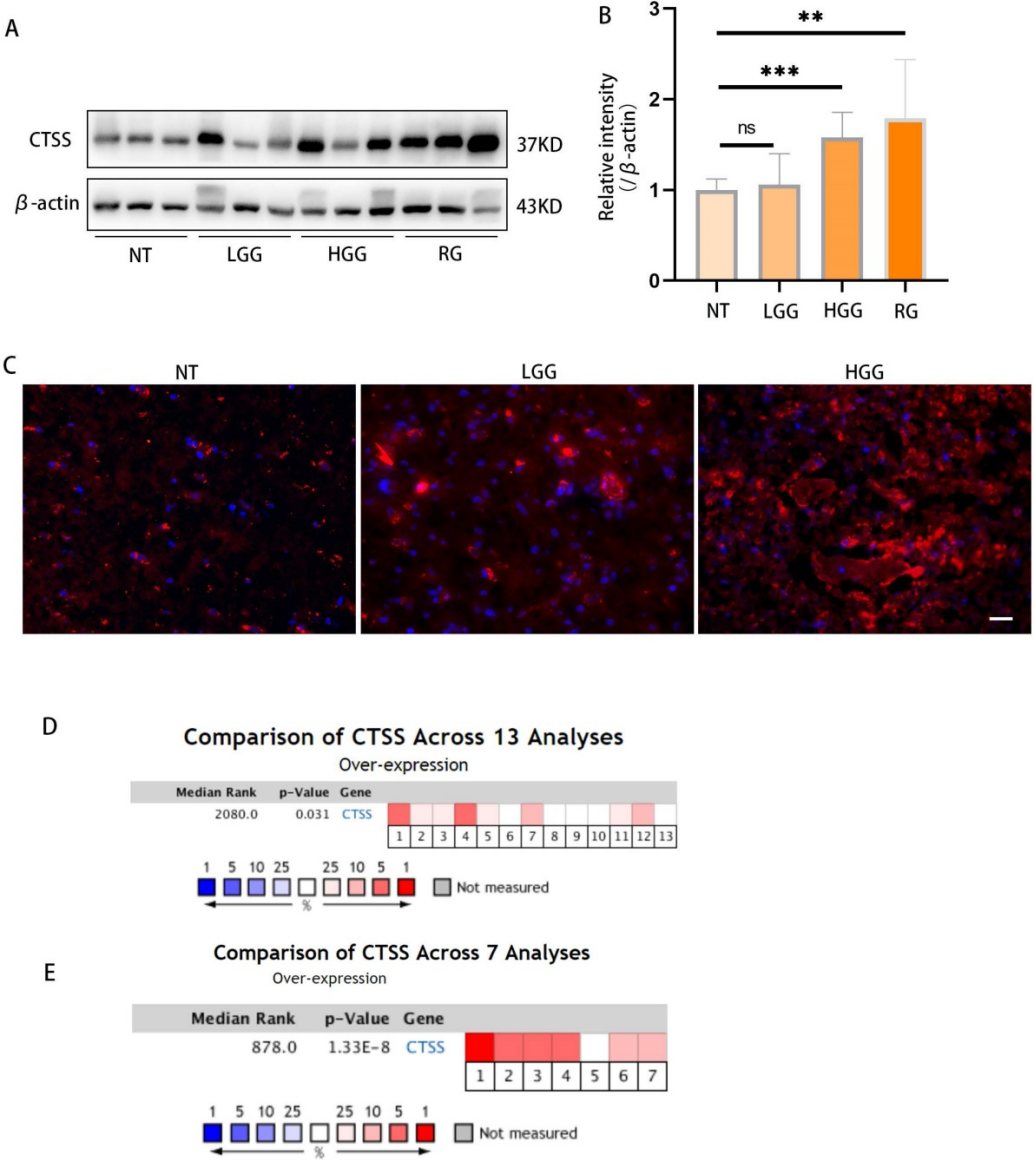
** Cathepsin S is highly expressed in GBM tissues.** (A, B) The levels of different grades of glioma tissues were detected by western blot. As shown here, CTSS was highly expressed in high grade glioma (p<0.001). (C) Immunofluorescence staining of CTSS was carried on the normal tissue, low grade glioma and high-grade glioma. CTSS was obviously overexpressed in the high-grade glioma. Scale bar, 25μm. (D) A meta-analysis of CTSS gene expression in low grade glioma from 13 Oncomine databases: Bredel Brain (1-3), French Brain (4,5), Gutmann Brain (6), Liang Brain (7), Rickman Brain (8), Shai Brain (9,10), Sun Brain (11-13). (D) A meta-analysis of CTSS gene expression in glioblastoma from 7 Oncomine databases: Bredel Brain (1), French Brain (2), Lee Brain (3), Liang Brain (4), Pomeroy Brain (5), Sun Brain (6), TCGA Brain (7). Data were represented as the means ± SEM of three independent experiments. *p<0.05, **p<0.01, ***p<0.001 versus control group.

Previous studies showed that CTSS was highly expressed in the human astrocytoma and glioblastoma tumor. Here, we confirmed this finding in glioma specimens by western blot and immunofluorescence staining (Fig. 1A-C). Meta-analysis via Oncomine showed that CTSS was highly expressed in 8 out of 13 experiments on glioma and 6 out of 7 experiments on glioblastoma through RNA-Seq (Fig. 1D, 1F) 10.

### ZFL inhibits TGF-β-induced EMT in glioblastoma cell lines

**Figure 2:**
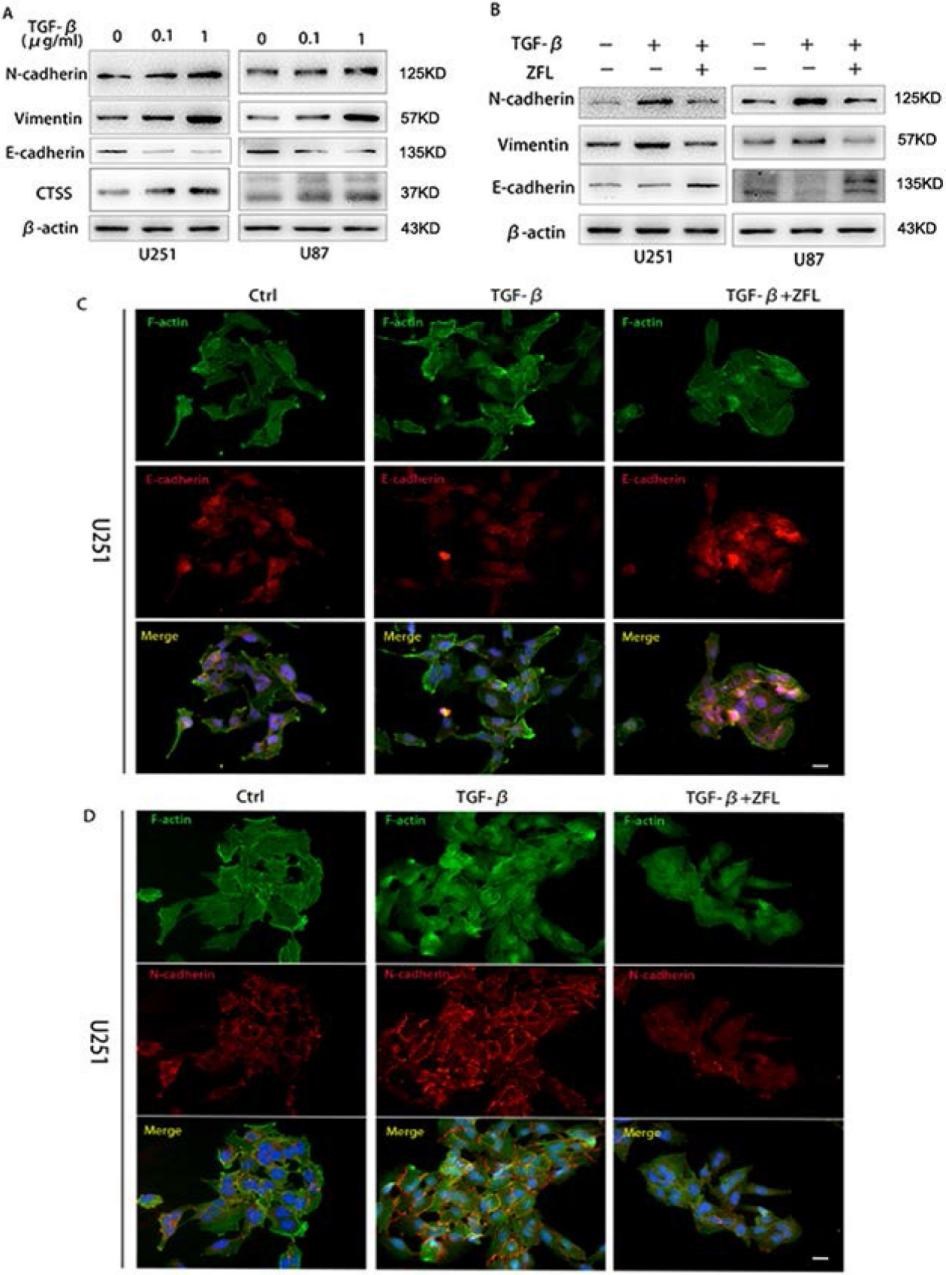
** CTSS inhibitor ZFL suppresses TGF-β induced EMT in glioblastoma cell lines.** Glioblastoma cell lines U251 and U87 were separately treated with 0, 0.1 µg/ml or 1 µg/ml TGF-β for 24 hours. (A) Western blot showed that the expression of CTSS was elevated with the dosage of TGF-βaccordingly. Meantime, mesenchymal proteins, N-cadherin and Vimentin, were also increased, while E-cadherin was dose-dependently decreased. (B) Further, cells were treated with 1 µg/ml TGF-β and ZFL was added in the TGF-β+ZFL groups. E-cadherin was increased while N-cadherin and Vimentin were obviously down-regulated in the TGF-β+ZFL groups. (C, D) Furthermore, immunofluorescence staining of E-cadherin and N-cadherin also showed the seesaw-like change of these two proteins. Scale bar, 25 µm.

Since TGF-β is one of the most important factors in tumor microenvironment and played a pivotal role in tumor mesenchymal transition, here we wondered whether TGF-β induced invasive growth and endothelial-mesenchymal state in glioblastoma and the potential role of CTSS in this process. As shown in Fig. 2A, TGF-β increased the expression levels of mesenchymal-related proteins such as N-cadherin and vimentin; besides, it decreased the expression of E-cadherin, an important marker of endothelial status, in U251 and U87 cell lines. Meanwhile, TGF-β augmented the expression of CTSS in a dose-dependent manner.

To investigate whether CTSS participated in the effect of TGF-β in glioblastoma cells, a CTSS inhibitor, ZFL (20 μM), was used in this experiment. By pharmacological inhibition of CTSS, we found that the expression levels of N-cadherin and Vimentin were down-regulated in the TGF-β+ZFL groups compared with TGF-β groups (Fig. 2B). On the contrary, E-cadherin was elevated in the TGF-β+ZFL groups (Fig. 2B). Additionally, immunofluorescence staining of E-cadherin and N-cadherin under colony status *in vivo* showed that N-cadherin was stained in a grid pattern and less staining spot of E-cadherin could be seen at the touching boundaries of cells after adding TGF-β (Fig. 2C, D). However, the staining of N-cadherin was disorganized, and the E-cadherin expression was observed when ZFL was added into the TGF-β-treated groups (Fig. 2C, D).

### ZFL decreases glioblastoma cell mobility and redistribute intermediate filament Vimentin under the context of TGF-β

**Figure 3:**
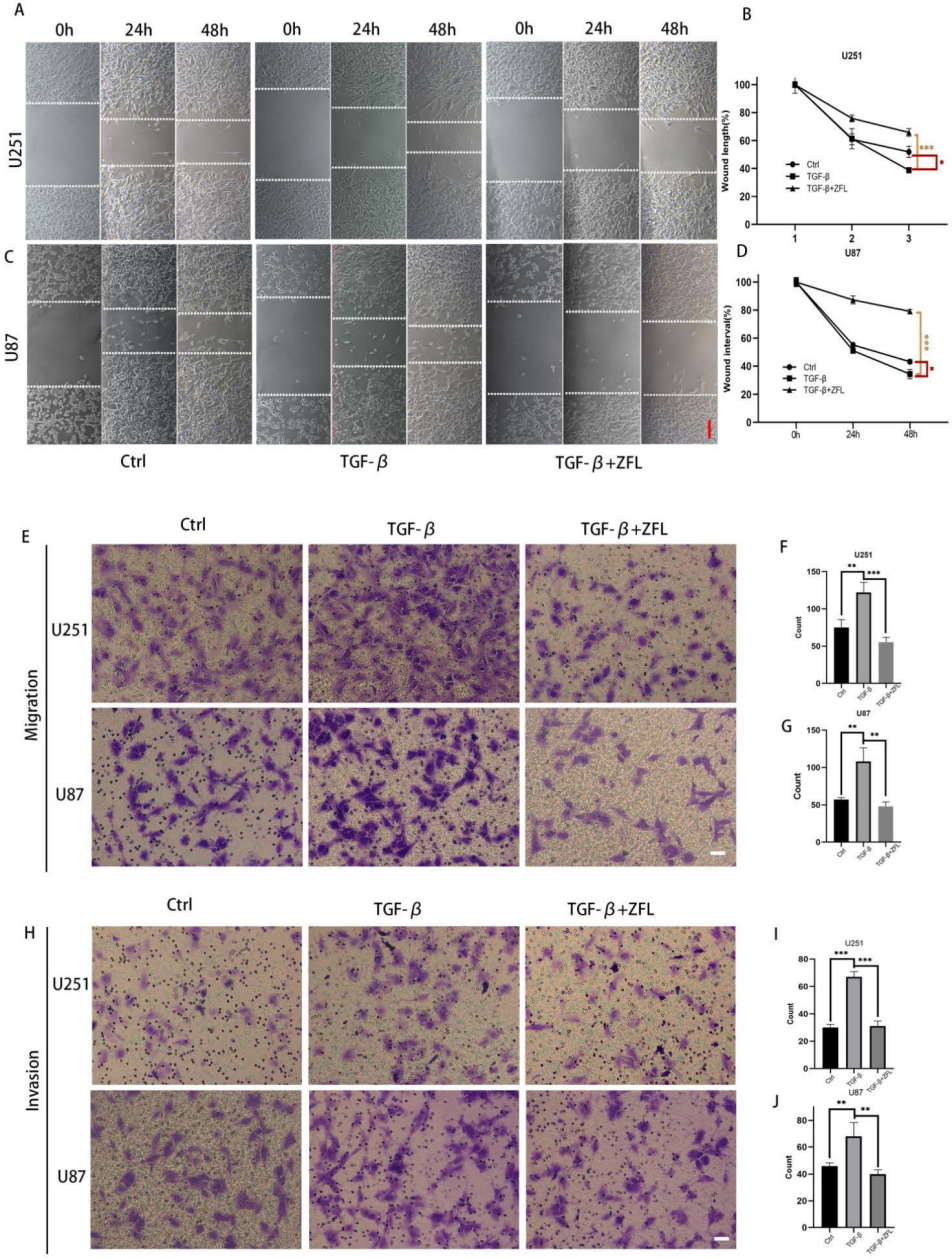
** ZFL decreases TGF-β-induced migration and invasion ability in glioblastoma cell lines.** (A) Full-fused cells with similar width of wound were prepared and then treated as above. The images of wound in different groups were recorded at 0 hour, 24 hours and 48 hours. Scale bar, 80μm. (B, C) The graphic analysis of wound healing speed was carried on by ANOVA analysis. (E-G) By migration assay, ZFL decreased TGF-β-induced migration in glioblastoma cell lines. Scale bar, 50μm. (H-J) By invasion assay, ZFL decreased TGF-β-induced migration in glioblastoma cell lines. Scale bar, 50μm.Data were represented as the means ± SEM of three independent experiments. *p<0.05, **p<0.01, ***p<0.001 versus control group.

Alongside with TGF-β-induced EMT, the migration rate in TGF-β treated groups was accelerated in the U251 and U87 cell lines (Fig. 3A-D). Transwell experiments also showed that more cells penetrated through the micropores after the usage of TGF-β, which indicated that TGF-β enhanced the metastasis and invasion ability of glioblastoma cell lines (Fig. 3E-J). However, ZFL could significantly decrease wound-healing speed in TGF-β-treated groups and retarded TGF-β-irritated metastasis and invasion ability (Fig. 3A-J).

**Figure 4:**
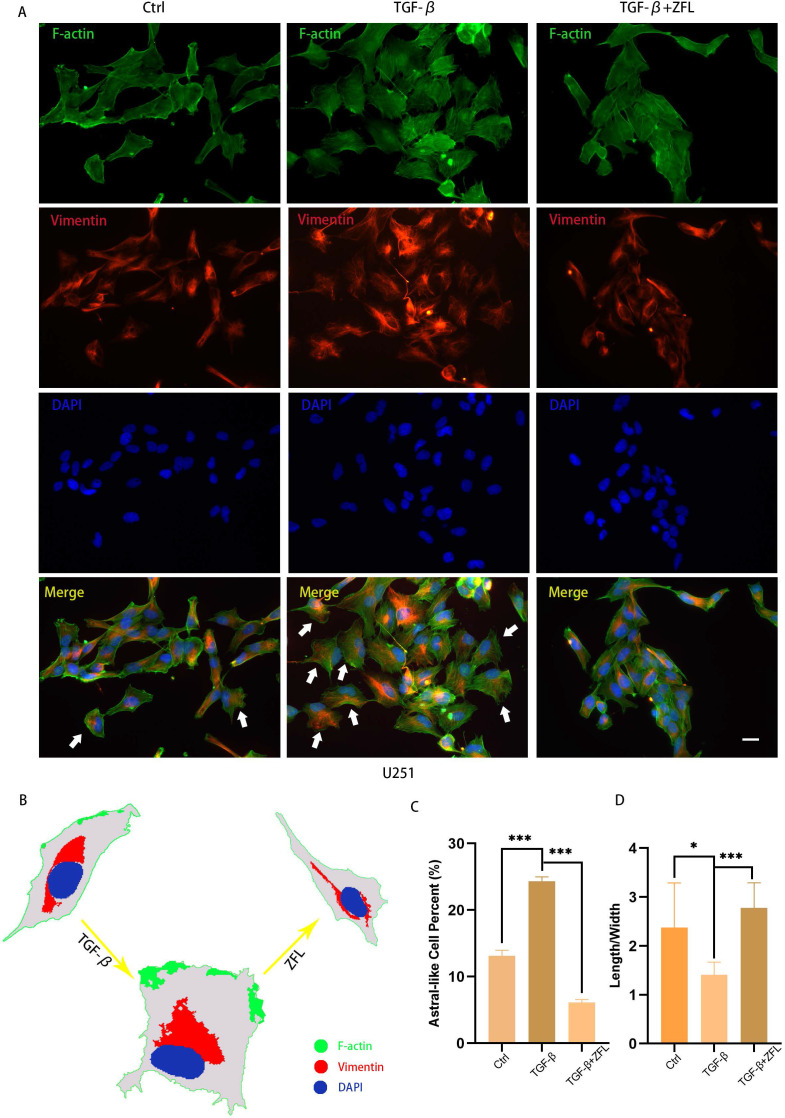
** ZFL restores TGF-β-induced morphological transformation and spatial change of Vimentin fiber in U251 cell line.** Cells were treated as above. (A) F-action and Vimentin were visualized separately on different groups. (B) Illustration mimicked the morphological change and Vimentin spatial change in U251 cell line. (C) The proportion of astral-like cells was calculated. Astral-like cells were significantly increased in the TGF-β group and decreased in the TGF-β+ZFL group (p<0.001). (D) Length to width ratio of 10 random cells was recorded in different groups. The Ratio of the control group, the TGF-β group and the TGF-β+ZFL group were 2.377±0.9118, 1.407±0.2590 (p<0.05, compared with the control group) and 2.774±0.5189 (p<0.001, compared with the TGF-β group), respectively. Scale bar, 25μm. Data were represented as the means ± SEM of three independent experiments. *p<0.05, **p<0.01, ***p<0.001 versus control group.

We also observed the morphological transition of U251 cell and spatial change of vimentin fiber in different groups by F-actin and Vimentin co-staining. The majority of glioblastoma cells displayed a longish shape to trapezoid-like shape with a length to width ratio of 2.377±0.9118 (Fig. 4A, 4G). Upon addition of TGF-β for 24 hours, more cells transformed from round to astral-like shape with a length to width ratio of 1.407±0.2590 (Fig. 4A, 4G), with nucleus para-shift and vimentin fiber polarity (Fig. 4A-C). However, after ZFL was added, most cells retained an oblong structure and the length to width ratio increased to 2.774±0.5189 (Fig. 4G), and vimentin fiber was distributed around the nucleus (Fig. 4A-C).

### Inhibition of CTSS restores TGF-β-induced tight junction turnover

**Figure 5:**
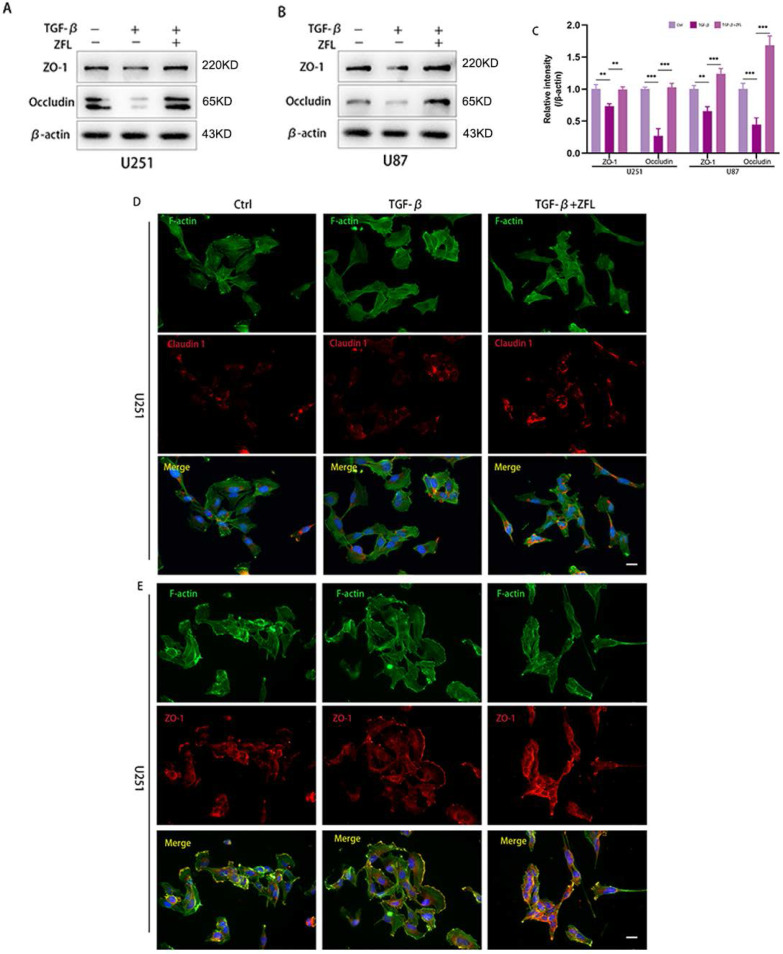
** Inhibition of CTSS restores TGF-β-induced tight junction turnover.** Cells were treated as above. (A-C) The expressions of the tight junction proteins, ZO-1 and Occludin, were detected by western blot. ZO-1 and Occludin were decreased in the TGF-β groups, while elevated in the TGF-β+ZFL groups. (D, E) Immunofluorescence staining of Claudin 1 and ZO-1 in different groups was also recorded. Scale bar, 25 µm. Data were represented as the means ± SEM of three independent experiments. *p<0.05, **p<0.01, ***p<0.001 versus control group.

Since loss of tight junction was highly related to tumor cells metastasis, hence, we detected the expression levels of some representative proteins of tight junction by western blot. As shown in the Fig. 5A-C, the markers of tight junction proteins, including claudin 1, ZO-1 and Occludin, were significantly decreased after TGF-β was added. However, ZFL could significantly elevated the expression levels of claudin 1, ZO-1 and Occludin when compared with the control group and TGF-β-treated group (Fig. 5A-C). Immunofluorescence staining of claudin 1 showed that claudin1-positive particles were sporadically observed in the dispersed cells of the control group and TGF-β-treated groups. However, claudin1-positive particles were commonly concentrated both in the separated cells and colonial cells after ZFL was added with TGF-β (Fig. 5D). What's more, immunofluorescence staining of ZO-1 indicated ZO-1 was strongly positive at the fused edge of cells in the TGF-β+ZFL group, but this relationship was relatively weak in the control group and TGF-β group (Fig. 5E).

### Inhibition of CTSS reverses TGF-β-induced EMT through suppressing the PI3K/AKT/mTOR pathway

**Figure 6:**
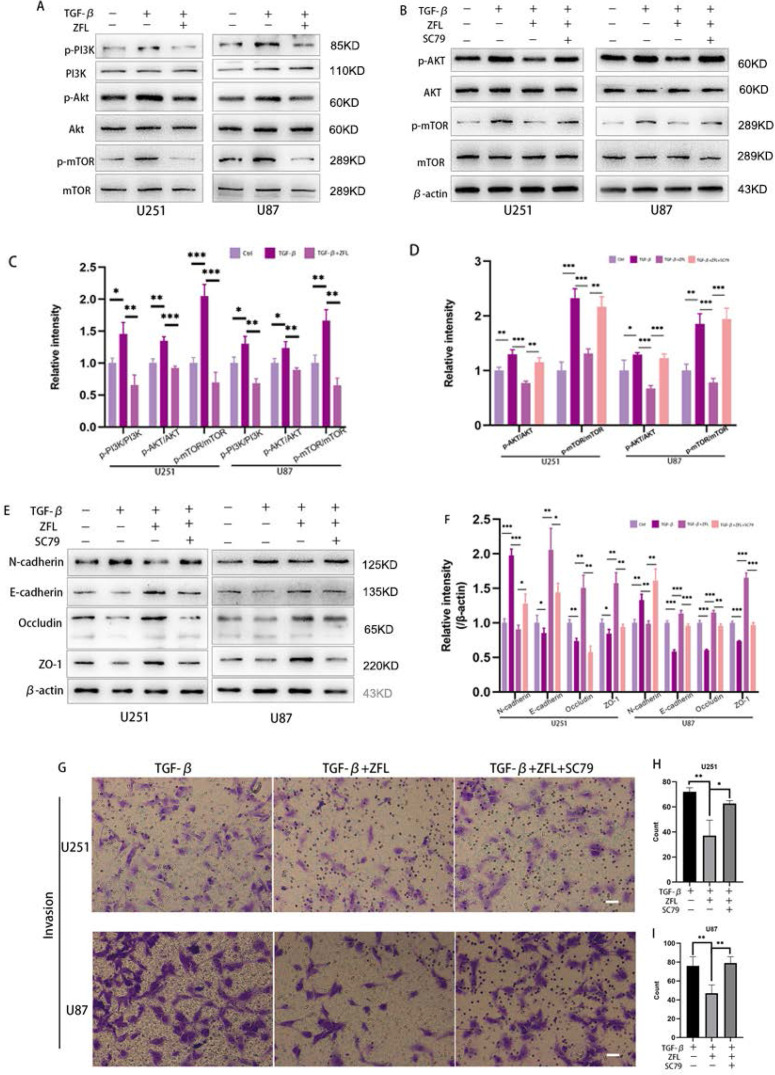
** Inhibition of CTSS reverses TGF-β-induced EMT through suppressing the PI3K/AKT/mTOR pathway.** (A, C) Cells were exposed to different treatments as above. The expressions of PI3K, p-PI3K, AKT, p-AKT, mTOR and p-mTOR were detected. (B, D) After adding AKT activator, SC79, the changes of AKT, p-AKT, mTOR and p-mTOR were detected. (E, F) After adding AKT activator, SC79, the changes of N-cadherin, E-cadherin, Occludin and ZO-1 in U251 and U87 cell lines were detected. (G-I) After adding AKT activator, SC79, invasion ability was detected by transwell. Scale bar, 50 µm. The relative protein levels of control cells were adjusted to the value of 1. Data were represented as the means ± SEM of three independent experiments. *p<0.05, **p<0.01, ***p<0.001 versus control group.

We used to find that inhibition of CTSS in glioblastoma cell lines could suppress the PI3K/AKT/mTOR pathway in previous study. Interestingly, studies have indicated that this pathway took an important role in TGF-β-irritated metastasis. Thus, we determined whether inhibition of CTSS reversed TGF-β-induced EMT through suppressing the PI3K/AKT/mTOR pathway. By western blot, TGF-β increased the expression levels of phosphorylation of PI3K, AKT and mTOR in the glioblastoma cell lines (Fig. 6A, C). However, ZFL could significantly decline the elevation of p-PI3K, p-AKT and p-mTOR (Fig. 6A, C), suggesting that the PI3K/AKT/mTOR pathway participated in the anti-EMT role of CTSS. To confirm these results, we used the AKT activator, SC79. As shown in Fig. 6B, SC79 could significantly increase the expression levels of p-AKT and p-mTOR. In addition, SC79 reversed the effect of ZFL on EMT through increasing mesenchymal-related proteins such as N-cadherin and vimentin, and decreasing the level of E-cadherin and related tight junction proteins (Fig. 6E, F). Furthermore, transwell experiments represented that more cells penetrated from upper well when SC79 was added into the TGF-β+ZFL groups (Fig. 6G-I).

## Discussion

Invasive growth as well as dispersed cancer cells around the tumor lesion is one of the most important features of glioblastoma, which may lead to the tumor recurrence after radiographically total resection. TGF-β, highly expressed in the tumor microenvironment, could facilitate tumor EMT and then contribute to migration. In the context of glioblastoma, TGF-β could be secreted by tumor cells and tumor associated macrophage and microglia. Previous studies showed that lysosomal cysteine proteases were involved in tumor metastasis in the TGF-β signaling. Recently, Zhang et al found that CTSL could take part in the TGF-β-induced EMT in A549 and MCF-7 cells 11. However, compared with CTSL, CTSS could remain catalytically active in both lysosomal acid environment and neutral context outside the lysosome 12. Moreover, CTSS was highly expressed in glioblastoma on the basis of our previous study 13, 14. CTSS could degrade extracellular matrix and further facilitate tumor migration 9. However, the role of CTSS in TGF-β-induced EMT in glioblastoma was remained unknown. Here, we demonstrated that inhibition of CTSS by ZFL could restore TGF-β-triggered EMT and disruption of tight junction through inhibiting PI3K/AKT/mTOR pathway.

The focus on the cysteine proteases' role of cancer progression was mainly placed on CTSB, CTSL, CTSD, and CTSS 7. Selective CTSS deficiency could impair angiogenesis and deter tumor proliferation through the regulation on the type IV collagen-derived anti-angiogenic peptides and bioactive pro-angiogenic gamma 2 fragments from laminin-5 15, 16, which indicated a fundamental role for extracellular matrix degradation by CTSS. Furthermore, other studies also demonstrated that inhibition of CTSS could trigger abnormality of tumor metabolism and subsequent apoptosis, which was highly related to its essential role in lysosome 17, 18. Besides, inhibition of CTSS reduced cancer cell invasion 19, 20. This phenomenon was observed in several studies and the reason might attribute to its role in ECM degradation 9, but the underlying mechanism was still vague. In our study, we found that TGF-β could increase the expression of CTSS in glioblastoma and, meantime, induce expression levels of mesenchymal proteins (N-cadherin, Vimentin), and decrease E-cadherin level in glioblastoma. However, CTSS suppression restored the change of TGF-β-induced cell morphology, increased the expression of E-cadherin and decrease N-cadherin and Vimentin. Except the expression level of the intermediate filament Vimentin, we also observed the change of Vimentin architecture around the nuclear. Recently, some researchers reported that vimentin fibers coaligned with the anisotropic orientation of traction stresses and were required for integrating and reorienting actin-based forces, thus permitting persistent single-cell polarity and migration 21, 22. The changes of intermediate filament Vimentin and F-actin indicated that CTSS could regulate single cell skeleton, and intrigue cancer cell transforming into a state with high movement ability.

In the current study, we analyzed that alongside with the change of EMT related proteins, tight junction proteins were altered after administration of CTSS inhibitor as well. The tight junction proteins are composed of integral membrane proteins and peripheral membrane proteins. As the apical structure of epithelial cells, tight junction could act as a gatekeeper for the paracellular diffusion of ions and certain molecules. Loss of tight junction proteins could impair integrity between cells, release single cell with high mobility and facilitate tumor invasion 23. Claudins, Occludin and zonula occludens (ZO) are mostly studied in cancer researches. Therefore, we mainly focused on the Claudin 1, Occludin and ZO-1. We found that the expressions of Claudin 1 and Occludin were significantly decreased in glioblastoma cells after treatment of TGF-β. Previous studies indicated that TGF-β could induce the loss of tight junction proteins in several types of cancers 24, 25. Interestingly, inhibition of CTSS restored the loss of ZO-1 and Occludin, and increased the level of Claudin 1. The role of Claudin 1 and Occludin in tumor progression and metastasis remained controversial. Recent literatures reported that Claudin 1 is up-regulated in several cancers, such as cervical cancer, colorectal cancer, gastric cancer and melanoma, and has a close relationship with cancer progression 26, while it is down-regulated in breast carcinoma, liver cancer, prostate cancer and glioblastoma and shows a tumor suppressive role^26^, ^27^. The opposite role of Occludin in different cancer was also reported 28-30. In view of tightly engaged relationship between tight junction turnover and EMT, further studies were needed to illuminate the detailed role of Claudin 1 and Occludin in MET change after inhibition of CTSS. Additionally, immunofluorescence staining showed that ZO-1 was mostly localized at the cell-cell adhesion membrane in the TGF-β+ZFL groups compared with the TGF-β groups. These data indicated that tight junctions could be regulated by CTSS but the specific mechanism needs to be further studied.

PI3K/AKT/mTOR pathway participates in a broad range of cancer regulatory processes, including cancer metabolism, proliferation and migration 31. Since PI3K/AKT/mTOR pathway was proved to play an crucial role in TGF-β-induced EMT 32, here we wondered whether inhibition of CTSS could influence this classic pathway. In the previous study, we found that the application of CTSS inhibitors in glioblastoma cell lines could down-regulate the activated state of PI3K/AKT/mTOR pathway 18. This phenomenon was also displayed under the context of TGF-β. Further, we used AKT activator SC79 to certify that activation of the PI3K/AKT/mTOR pathway could reverse the decreased ability of invasion by CTSS inhibitor. The results showed that SC79 up-regulated N-cadherin, vimentin and down-regulated E-cadherin and tight junction proteins, indicating that inhibition of CTSS restored TGF-β-triggered EMT and disruption of tight junction through inhibiting PI3K/AKT/mTOR pathway.

PI3K/AKT/mTOR pathway is usually activated in various tumors, meanwhile, activating mutation of the PI3K genes (commonly the PIK3CA gene) were frequently seen in human cancers. Inhibition of this pathway *in vitro* could acquire anti-proliferative and anti-metastatic effects. However, the clinical trials about application of PI3K inhibitor did not show satisfactory results partly due to its adverse effect such as systemic toxicity 33. Compared with PI3K inhibitors, suppression of CTSS not only decreased the PI3K signaling but also impaired the process of tumor angiogenesis and tumor growth. Therefore, it is promising for further studies on CTSS inhibitors in glioblastoma and other cancers.

## Conclusion

Taken together, our results demonstrated that inhibition of CTSS could reverse TGF-β-mediated EMT and tight junction proteins turnover in human glioblastoma cell lines through suppressing the PI3K/AKT/mTOR pathway.

## Abbreviations

CTSS: cysteine protease, cathepsin S
ZFL: Z-FL-COCHO
TGF: transforming growth factor
ECM: extracellular matrix
ZO: zonula occludens
